# One‐Step Reconstruction with a Novel Suspended, Modular, and 3D‐Printed Total Sacral Implant Resection of Sacral Giant Cell Tumor with Preservation of Bilateral S_1–3_ Nerve Roots *via* a Posterior‐Only Approach

**DOI:** 10.1111/os.12582

**Published:** 2019-12-18

**Authors:** Zhao‐rui Lv, Zhen‐feng Li, Zhi‐ping Yang, Xin Li, Qiang Yang, Ka Li, Jianmin Li

**Affiliations:** ^1^ Department of Orthopedics Qilu Hospital of Shandong University Jinan China; ^2^ Cheeloo College of Medicine Shandong University Jinan China

**Keywords:** 3D‐printed total sacral implant, Giant cell tumor of the sacrum, Modular, One‐step sacral reconstruction, Suspended, Total piecemeal resection

## Abstract

**Objective:**

To investigate the efficacy and safety of spinopelvic reconstruction based on a novel suspended, modular, and 3D‐printed total sacral implant after total piecemeal resection of a sacral giant cell tumor (SGCT) with the preservation of bilateral S_1–3_ nerve roots *via* a posterior‐only approach.

**Methods:**

Five patients who had undergone total piecemeal resection of SGCT involving upper sacral segments (S_1_ and S_2_) and the midline with the preservation of bilateral S_1–3_ nerve roots *via* a posterior‐only approach between September 2017 and July 2018 were retrospectively reviewed. A novel suspended, modular, and 3D‐printed total sacral implant had been used for reconstruction. This series included two female and three male patients, with a mean age of 42.2 years (range, 31–53 years). Surgical time, blood loss, complications, preoperative and postoperative neurological function, instrumentation failure, and local control were presented and analyzed.

**Results:**

All patients underwent the operation without death or serious complications. The implant was installed on the defect, connecting the ilium and lumbar vertebrae, and fixed with a screw–rod system up to the level of L_3–4_ or L_4–5_. The mean operative time was 502 min (range, 360–640 min) and the mean operative blood loss 4400 mL (range, 3000–7000 mL). The mean follow‐up was 15 months. After the operation, pain was significantly relieved, and the patients resumed walking as early as 2 weeks later. The patients showed no neurogenic bladder dysfunction and no fecal incontinence or gait disturbance. Wound healing was poor in one patient. Patients recovered well without evidence of local recurrence. No implant failures or related clinical symptoms were detected during follow up. Satisfactory bone ingrowth and osseointegration at the bone‐implant junctions was found in follow‐up CT.

**Conclusion:**

Although technically challenging, it is feasible and safe to use a suspended, modular, and 3D‐printed implant for reconstruction after total piecemeal resection with the preservation of bilateral S_1–3_ nerve roots in patients with SGCT. We believe that this implant can be applied to sacral reconstruction in a wide variety of diseases.

## Introduction

Giant cell tumor of the bone is a locally aggressive benign bone tumor that rarely metastasizes to the lungs, with malignant transformation occurring in fewer than 1% of all giant cell tumors of the bone (GCTB)^1^. Sacral giant cell tumors (SGCT) have no specific symptoms in the early stages and are often quite large at diagnosis. Patients present with lower back pain, frequently radiating to the legs, and sometimes with bladder, rectal, or sexual dysfunction. Surgical resection is necessary to improve local control and survival for patients with giant cell tumors of the bone. Surgical management of SGCT is challenging because of the large size of the tumors, massive bleeding, spinal instability, and involvement of sacral nerve roots. Cortical destruction and soft tissue extension by expansive tumor growth are common. Some giant cell tumors (GCT) involve the upper sacral segments, frequently crossing the midline and even the sacroiliac joint. Definitive treatment guidelines for SGCT have not been established^2^. The optimal treatment of SGCT remains controversial^3^. Although surgery with wide margins results in a significant decrease in the local recurrence rate, wide resection often requires the sacral nerve roots to be sacrificed and there is significant risk of postoperative neurological damage^4^. Total piecemeal resection is a viable alternative if total en bloc resection, which is associated with an excellent prognosis, is unfeasible.

Surgery for giant cell tumors involving the S_1_ vertebra often requires spinopelvic reconstruction. When S_1_ remains incomplete after resection, weight‐bearing capacity is considered insufficient and reconstruction needs to be performed. Spinopelvic reconstruction is challenging in terms of anatomical complexity, excessive load, and extensive defects, and there is a high risk of complications. The pedicle iliac screw rods system with human bone or titanium mesh is typically used to rebuild biomechanical stability of the spinopelvic complex. The previous spinopelvic reconstruction method is often unable to reconstruct defects satisfactorily. Mechanical failure, such as rod fatigue fracture and loosening, is sometimes encountered^5^, ^6^. Mechanical failure occurred in 16.1%–25% of patients undergoing spinopelvic reconstruction^7^, ^8^. Effective reconstruction of the spinopelvic continuity is important because it allows the patient to walk early, reducing postoperative complications.

The technological advances in 3D‐printing may help us overcome these difficulties. 3D‐printed technology has been successfully used in bone tumor surgery during preoperative planning, resection, and reconstruction^9^. 3D‐printed technology is ideal for fabricating a custom‐made implant with internal porous structures that enhance osseointegration at the bone–implant junctions. A few cases have been reported of spinopelvic reconstruction with a 3D‐printed implant in sacral malignant tumor surgery accompanied by the sacrifice of the sacral nerve roots^10^, ^11^, ^12^. With the blockade of the preserved nerve root, the previously used one‐piece implant is difficult to place. In this paper, to overcome these limitations, we describe a novel suspended, modular, and 3D‐printed total sacral implant. The purposes of the article are to investigate the efficacy and safety of reconstruction using this implant after total piecemeal resection of SGCT with the preservation of bilateral S_1‐3_ nerves *via* a posterior‐only approach. To our knowledge, there are no previous case series reports of successful spinopelvic reconstruction using a 3D‐printed total sacral implant in patients with SGCT after tumor resection.

## Materials and Methods

### *Patients*

Five patients who had undergone total piecemeal resection of SGCT involving upper sacral segments and the midline with the preservation of bilateral S_1–3_ nerves *via* a posterior‐only approach between September 2017 to July 2018 were retrospectively reviewed. Suspended, modular, and 3D‐printed total sacral implants were used for reconstruction. There were three men and two women in this case series, with a mean age at the time of diagnosis and admission of 42.2 years (range, 31–53 years). Informed consent was obtained from all individual participants included in the study. The protocol for the research project was approved by the Medical Ethics Committee of Qilu Hospital of Shandong University and it conforms to the provisions of the Declaration of Helsinki (as revised in Brazil in 2013). Patient characteristics and outcomes are provided in Table 1.

**Table 1:** Patient characteristics and outcomes

Casenumber	Age (years), Sex	Level	Size (cm)	Blood loss (mL)	Surgical time (min)	Spared level of nerve root	Complications	Preoperative neural status	Postoperative neural status function scoring			Follow ‐up(month)
									Motor function and sensation of lower limbs	Urination and uriesthesia	Defecation and rectal sensation	
1	35，M	S_1–__4_	10	7000	540	Bilateral S_3_	None	Pain, swelling	7	8	8	21
2	31，M	S_1–__3_	10	3500	640	Bilateral S_3_	Impaired wound healing	Pain	9	9	8	17
3	51,M	S_1–__3_	6	3500	360	Bilateral S_3_	None	Pain, sciatica, motor deficit, neurogenic bowel & bladder	7	8	8	17
4	41,F	S_1–__4_	8	3000	570	Bilateral S_3_	None	Pain	8	8	8	16
5	53,F	S_1–__3_	10	5000	400	Bilateral S_3_	None	Pain, sciatica, neurogenic bowel	8	7	8	13

### *Imaging Studies*

All patients underwent plain radiography, CT, and MRI to identify the form, location, and size of the tumor and its relationship with surrounding structures. All tumors were located in the S_1_ vertebrae and below. S_1–4_ were involved in two patients; S_1–3_ were involved in three patients; MRI revealed presacral soft tissue masses in four patients.

### *Preoperative Biopsy*

A preoperative biopsy was planned to obtain tissue for a pathological diagnosis after imaging studies. Percutaneous CT‐guided needle biopsy is the preferred diagnostic technique in these cases, not only yielding adequate tissue for diagnosis but also minimizing the possibility of tumor cell contamination. All patients underwent CT‐guided needle biopsy and histologic examination showed giant cell tumors of the bone.

### *Denosumab*

Denosumab was administered subcutaneously at a dose of 120 mg twice a month before surgery under oral supplementation of calcium and vitamin D. Subjective symptoms were relieved after treatment of neoadjuvant denosumab in all five patients. The tumors were reduced in size and clear in the boundary.

### *Preparation of Sacral Implant*

**Figure 1:**
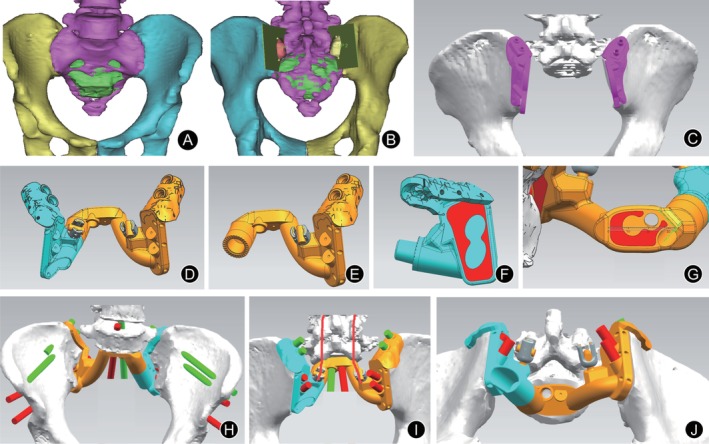
Design of sacral implant and guides. (A) A 3D bone tumor model was created for surgical planning. The 3D pelvis showed the extent of the tumor (green in color). (B) Surgeons performed the virtual resections by defining the locations and orientations of the resection planes. (C) The design of osteotomy guide plates had: the cutting platforms that matched the planned resection planes; the flanges with the contoured shape that allowed osteotomy guide plates positioning on the surgically accessible bone surface decided by the surgeons using the computer‐aided design software; and the K‐wire holes on the flanges for stabilizing the osteotomy guide plates to the bone. (D) 3D implant model. The implant consisted of two modules that are connected by a sleeve device with serrated teeth locked by a screw. (E)The first module. (F)The second module with a porous structure (red in color). (G) The proximal surface with porous structure (red in color) of the center part fitting to the inferior endplate of the L_5_ vertebrae. The 3D pelvic model ((H) front view, (I) dorsal view and (J) bottom view) showed the implant contacting the surface of the L_5_ inferior endplate, ilium osteotomy planes, and the iliac crest. Screw positions and lengths were planned, based on the bone thickness and quality of the remaining bone after resection. Screw direction was in accordance with the direction of mechanical transmission.

**Figure 2:**
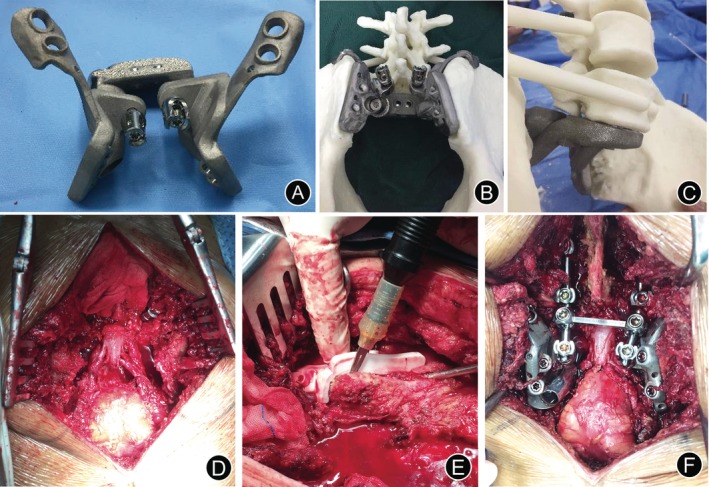
Preoperative simulation and intraoperative images. (A–C) The outer view of the implant. These models included the implant trial and the remaining bone after tumor resection allowed the surgeon to practice the procedures before the real surgery. (D) The intraoperative image showed the bone defect between L_5_ and both sides of the ilium after resection of the tumor. The bilateral S_1–3_ sacral nerves were dissected and preserved. (E) The intraoperative image showed that the osteotomy guide plates helped us complete the iliac bone osteotomy bilaterally. (F) The intraoperative photo showed that the implant was settled.

The sacral implant was designed as a patient‐specific structure (Fig. 1). Pelvic CT was performed to identify details of the anatomical location and the structure of the lesion; axial images were reconstructed at 1.0‐mm slices with 1.0‐mm slice spacing. CT images were processed in DICOM format and were exported to the software MIMICS (Materialize, Leuven, Belgium) to reconstruct a 3D rendering (Fig. 1A). After osteotomy planes were determined (Fig. 1B), the customized implant was designed using 3D design software UNIGRAPHICS NX (Siemens PLM Software, Texas, USA) according to the shape of the bone defect (Fig. 1D). Then, the porous structure of the implant was designed using MAGICS software (Materialize, Leuven, Belgium). The implant consisted of two modules that are connected by a sleeve device with serrated teeth locked by a screw. The first module (Fig. 1E) consists of two parts, including a center part (Fig. 1G) contacting the surface of the L_5_ inferior endplate and a right wing‐part designed to connect the right ilium osteotomy plane and the right iliac crest. The second module (Fig. 1F) is a left wing‐part designed to connect the left ilium osteotomy plane and the left iliac crest. The ends of the two wing‐parts form inverted *U*‐shaped structures hooking the bilateral iliac crest which are fixed by two cancellous bone screws through two nail paths. There are two multiaxial screw heads situated on the back of the implant for connection to lumbar vertebrae with titanium rods. The implant consisted of three bone–implant junctions, including the proximal surface of the center part fitting to the inferior endplate of L_5_ vertebrae and the surfaces on both sides of the implant matched to the osteotomy planes of the bilateral iliac and iliac crest (Figs 1 and 2). Bone–implant junctions were of porous structure to facilitate ingrowth and were firmly fixed with bone using lock screws and/or cancellous bone screws through nail paths. The small holes on the backside of the implant are designed for soft tissue suture fixation (Fig. 1D). A metal 3D printer system (EOS M290, EOS GmbH Electro Optical Systems, Munich, Germany) was used to print the implant. Selective laser melting was used in fabrication by successive layering of melted titanium alloy according to a computer‐aided design model. The material was Ti‐6Al‐4V medical‐grade powder. The diameter of pores was 300 μm–700 μm, with an average porosity of 40%–80%. The implant was tested according to the National Standard of Implants for Surgery in China and was examined and modified several times.

### *Preparation of Guides*

The osteotomy guide plates (Fig. 1C), accurately indicating the range of osteotomy required for resection of tumors, were designed using 3D design software UNIGRAPHICS NX (Siemens PLM Software, Texas, USA). They were prepared based on the patient's anatomy of the ilium and were highly conformed to the surface morphology of bone and had positioning holes for Kirschner wire drilling. The data for the designed guides were imported into a 3D printer (Model: UP BOX, Tiertime, China) for printing. Polylactic acid (Tiertime, China) was used as a raw material. The customized guides were sealed and sterilized with ethylene oxide for intraoperative use.

### *Hemostasis*

An abdominal aorta balloon implantation was performed on the day of operation. The balloon was filled by injecting saline to temporarily block the abdominal aorta to reduce blood loss during the operation. Each block lasted for up to 50 min, with 15 min between each block.

### *Outcome Measures*

The outcome of postoperative neural status function was assessed at final follow‐up using a scoring system^13^ for evaluating neurological deficit after sacral resection which contains nine items and allots 0, 1, 2, or 3 points to each item according to the degree of functional deficits, with a maximum obtainable score of 27 points. These nine items are assigned to three categories: motor function and sensation of lower limbs, urination and uriesthesia, and defecation and rectal sensation (Table 1).

## Results

Treatment decisions were made by a multidisciplinary team of surgeons, pathologists, radiologists, and clinical oncologists. With the emergence of new technologies, treatment methods continue to develop, but our treatment objective was always based on a combination of maximizing tumor control and minimizing morbidity. Patient choice was a key factor in many decisions that were made.

### *Surgical Technique*

#### *Step 1: Exposure*

The operations were performed through a posterior‐only approach. The patient was placed prone on the operating table and an inverted Y incision was made. The para sacral muscles were dissected subperiosteally to expose the dorsal surface of the sacrum and coccyx, bilateral sacroiliac joints, part of the iliac bone, and L_3–5_ spinous process. Four pedicle screws were placed into the L_3–4_ or L_4–5_ pedicles. We performed a laminectomy and exposed the sacral nerve carefully. The ligaments around the sacrum were cut off and the sacrum was pulled back to expose the rectum. The tumors were bluntly separated from the rectum with gauze packed into the pre‐sacral space. Attention should be paid to ensuring the integrity of the intestinal wall.

#### *Step 2: Osteotomy and Piecemeal Resection*

Piecemeal resection of tumors was conducted. The bilateral S_1–3_ sacral nerves were dissected and preserved (Fig. 2D). The S_4–5_ nerve root and dural sac were cut and ligated. A temporary fixator was installed to maintain a normal lumbosacral anatomical position. The osteotomy guide plates were placed in the corresponding area to help us complete the iliac bone osteotomy bilaterally (Fig. 2E). Tumor and bilateral partial iliac bone were removed with a satisfactory margin. L_5_–S_1_ intervertebral discs were excised. We carefully checked whether there was any residual tumor and stopped the bleeding adequately.

#### *Step 3: Reconstruction*

The first module was placed through the space between the right L_5_ and S_1_ nerves and matched the right osteotomy planes of ilium and L_5_ inferior endplate. The second module was placed through the space between the left L_5_ and S_1_ nerves and matched the left osteotomy planes of the ilium. We fine‐tuned the sleeve device that connects the two modules to obtain a precise match. Screws were installed into vertebrae of L_5_ and the bilateral ilium through reserved nail road. Two rods were used to connect the pedicle screws and implant. (Fig. 2F).

### *Postoperative General Condition*

All five patients underwent total piecemeal resection of SGCT with preservation of the bilateral S_1–3_ nerves *via* a posterior‐only approach in one stage. The 3D‐printed customized guides based on CT data were successfully manufactured. All patients underwent the operation without death or serious complications. The implant was installed on the defect, connecting the ilium and lumbar vertebrae, and fixed with a screw–rod system up to the level of L_3–4_ or L_4–5_. The mean operative time was 502 min (range, 360–640 min) and the mean operative blood loss was 4400 mL (range, 3000–7000 mL).

### *Histologic Findings*

The excised specimens were sent to the pathology department for histologic analysis. The pathologic diagnoses were GCTB.

### *Complications*

**Figure 3:**
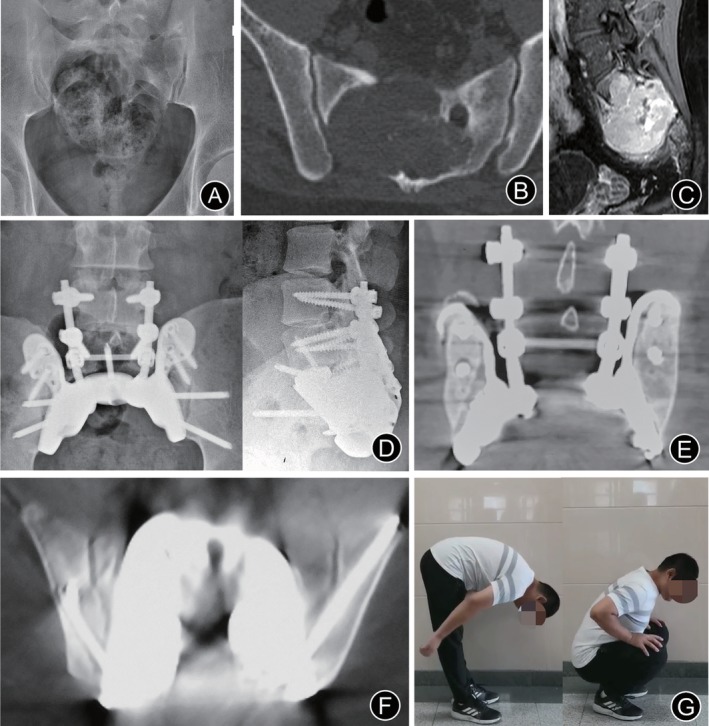
Imaging of the representative case. Preoperative X‐ray (A), axial CT (B), and contrast‐enhanced sagittal MRI (C) revealed a large soft tissue mass located in the upper sacrum. Postoperative X‐ray (D), and coronal (E) and axial (F) CT showed good implant alignment, no evidence of implant loosening, excellent bone ingrowth, and osseointegration at the bone–implant junctions in the 17 months after surgery. (F) He could squat and bend like a normal person.

There were no intraoperative complications. Poor wound healing occurred in one patient, who recovered completely after 2 weeks’ management with debridement and dressing change. The average duration of follow‐ up was 17 months, with a range of 13–21 months. No local recurrence or instrumentation failure was detected during follow up. After the operation, the pain was significantly relieved, and the patients resumed walking as early as 2 weeks later. The patients showed no neurogenic bladder dysfunction, fecal incontinence, or gait disturbance. Patients recovered well during the follow‐up period. Satisfactory bone fusion was found in CT (Fig. 3E, F).

### *Postoperative Management*

Physical examination, local X‐ray, local CT, and local MRI are performed every 3 months for the first 1 year after surgery and every 6 months thereafter. Chest CT was performed every 6 months for the first 2 years after surgery, and every year thereafter. The patients were assessed for tumor recurrence, urination and defecation function, lower limb sensation, myodynamia, and wound healing. We recommend short‐term use of denosumab after surgery. Not all patients were treated with radiotherapy or embolism.

### *Representative Case*

A 30‐year‐old man (case 2) complained of low back pain for 1 month. Anteroposterior radiographs of the sacrococcygeal area showed a large area of osteolytic destruction at the S_1_–S_3_ levels (Fig. 3A). CT showed that the tumor involved the right sacroiliac joint (Fig. 3B). MRI showed that the tumor involved the S_1_–S_3_ levels with an anteroposterior diameter of 10 cm (Fig. 3C). Total piecemeal resection *via* a posterior‐only approach was performed, and the bilateral S_1_–S_3_ nerve roots were retained. Poor wound healing occurred postoperatively and was treated by debridement and dressing change. No instrumentation failure was detected by X‐ray during follow up (Fig. 3D). Satisfactory bone fusion was found in CT (Fig. 3E, F). At 17‐month follow‐up, bowel and bladder functions were nearly normal, and lower extremity function was normal. The patient could squat and bend like a normal person (Fig. 3G). There was no recurrence during follow‐up.

## Discussion

### *Total Piecemeal Resection*

Surgical treatment remains the mainstay of treatment for SGCT, and recurrence is a major concern in treatment, likely because of the complex location and the large size before diagnosis^3^, ^14^. One of the core issues in the treatment of sacral tumors is the preservation of the sacral nerve roots. Earlier studies showed that bilateral preservation of the S_3_ nerve and above is necessary to maintain good mental health, physical health, bowel function, and sexual function^2^, ^14^, ^15^, ^16^. For malignant tumors, such as chordoma or osteosarcoma, nerve root sacrifice should be considered due to the infiltrative nature of the tumor. In contrast, when resecting benign tumors, nerve root preservation may be feasible. Guo *et al*. treated SGCT with intralesional curettage or partial excision and the 5‐year local recurrence rate was 20.4%^14^. This conservative surgery achieved satisfactory local control with the preservation of major function^17^. Total piecemeal resection can preserve the sacral nerves and may prevent or lessen these complications. Although total piecemeal resection is associated with the possibility of contamination of tumor cells in the surgical area, it is sometimes used to remove lesions to the greatest extent. In the present cases, the S_3_ nerve roots and above were preserved and no patients had experienced local recurrence during the follow up. The neurologic outcome of the present study is in accordance with the results reported in the literature^14^, ^15^, ^16^, ^18^, ^19^. Larger studies are encouraged to ascertain the efficacy of variable management approaches. It has been reported that resection and reconstruction using a posterior‐only approach are feasible and safe for patients with sacral tumors^20^, ^21^. The posterior‐only approach has been widely used for sacral tumors in our center, and all of the five patients in the present study underwent a posterior‐only approach without serious complications. Extensive hemorrhage is a serious complication during SGCT resection. Aortic balloon occlusion decreased blood loss volumes^22^.

### *Spinopelvic Reconstruction*

The bone defect caused by the total piecemeal resection of SGCT involving upper sacral segments (S_1_ and S_2_) and the midline often results in a disconnection between the pelvis and the lumbar spine that is similar to total sacrectomy. Total sacrectomy without reconstruction is associated with some problems in patients, such as being bedridden long term, nerve root distraction from the submergence of lumbar vertebrae, and walking disability. Therefore, spinopelvic reconstruction after total sacrectomy is recommended by several authors^23^, ^24^. Despite decades of effort, results using the spinopelvic reconstruction method are unsatisfactory and the procedure remains controversial. Tang *et al*. reported that postoperative fixation mechanical failure occurred in 25% of patients (16/63) who underwent total sacrectomy^8^. The conventional spinopelvic reconstruction techniques can be divided into three categories: spinal pelvic fixation^20^, ^25^, ^26^, posterior pelvic ring fixation^27^, ^28^, and anterior spinal column fixation 12. A biomechanical study and a systemic review show that a reliable reconstruction method should include all three categories^7^, ^29^. The 3D‐printed implant has been successfully used in bone tumor surgery^9^. The 3D‐printed total sacral implant provides a new concept for resection and spinopelvic reconstruction. There are a few case reports on reconstruction using custom‐made implants after resection of sacral tumor which are accompanied by the sacrifice of the sacral nerve roots^10^, ^11^, ^12^. With the blockade of the preserved nerve roots, the previously used implant is difficult to place.

### *Advantages of 3D‐Printed Implants*

This novel suspended, modular, and 3D‐printed total sacral implant can overcome the limitations. It integrates the three categories mentioned above simultaneously, which shortens the operation time and simplifies the procedure. The structure can suit the complex and high‐strength mechanical environment of the lumbosacral region. The modular design prevents nerve root damage in implant placement, avoiding excessive traction of the nerve roots. It also allows fine‐tuning when the implant does not match the actual bone defect precisely. The suspended design of the implant and screw direction in accordance with the direction of mechanical transmission enables the best mechanical distribution. The combination of a cancellous bone screw and a locking screw ensures the tight fit of the bone–implant junctions, ensuring the short‐term stability. By reducing the gap between the implant and the bone and increasing the contact area, tightly bonded implants can better maintain stability and reduce postoperative pain. The design of foramen around the implant is beneficial to the suture and fixation of soft tissue. The bone–implant junctions of the 3D‐printed implant are porous structures, which are beneficial to bone ingrowth and osseointegration, ensuring long‐term stability. The follow‐up results showed that excellent bony union can be achieved on both the densely structured strut surface and the loosely structured porous mesh.

### *Denosumab*

Denosumab, a fully human monoclonal antibody that inhibits RANKL, was approved by the Food and Drug Administration in 2013 and represents a new treatment option for GCT. The effect of preoperative denosumab therapy was confirmed for patients with unresectable GCTB and those with GCTB who needed surgical downstaging because surgery would be associated with severe morbidity^30^, ^31^, ^32^. For patients with resectable GCTB, neoadjuvant denosumab therapy resulted in beneficial surgical downstaging^33^. Preoperative denosumab treatment tends to reduce blood supply and blood loss of SGCT^34^. Less blood loss can mean there is a clear visible surgical field and the surgeon is better able to remove the tumor thoroughly. However, the efficacy of preoperative denosumab for GCTB is still controversial. Up to now, there has been no relevant report on denosumab therapy for SGCT after surgery. We recommend short‐term use of postoperative denosumab for the removal of possible contamination of tumor cells. The effect requires further study.

### *Limitations*

The present study has some limitations. First, this was a retrospective study, although it is, to our knowledge, the largest series to data on 3D‐printed total sacral implants for reconstruction after resection of SGCT. Second, the total number of subjects is small because of the relative rarity of SGCT, and this limits the power of statistical analysis and tests. Third, our follow up is too short to provide data concerning long‐term tumor control. In future studies, these findings may need to be confirmed with larger populations from multiple centers.

### *Conclusion*

The suspended, modular, and 3D‐printed total sacral implant is a reasonable choice for reconstruction after total piecemeal resection of SGCT with the preservation of bilateral S_1–3_ nerve roots. Considering the complexity of the surgery, we recommend that this surgery should be conducted by an experienced team with multidisciplinary cooperation and careful preoperative planning, which are key to success. We believe that this implant can be an effective means of managing sacral reconstructions in a wide variety of diseases.
